# Pattern and Outcome of Acute Non-ST-Segment Elevation Myocardial Infarction Seen in Adult Emergency Department of Al-Shaab Teaching Hospital: A prospective Observational Study in a Tertiary Cardiology Center

**DOI:** 10.7759/cureus.17981

**Published:** 2021-09-14

**Authors:** Mohamed Abdelhameed, Omran Hakim, Awad Mohamed, Eyad Gadour

**Affiliations:** 1 Emergency Medicine, Bahri Teaching Hospital, Khartoum, SDN; 2 Emergency Medicine, Khartoum North Hospital, Khartoum, SDN; 3 Cardiology, Alshaab Teaching Hospital, Khartoum, SDN; 4 Gastroenterology and Hepatology, University Hospitals of Morecambe Bay NHS Foundation Trust, Lancaster, GBR

**Keywords:** cardio protection, cardiovascular medicine. hypertensive heart disease. cardio-oncology. cardiac mri, primary pci, non-st elevated myocardial infarction, acute myocardial injury

## Abstract

Background

Despite investments to improve the quality of emergency care for patients with acute myocardial infarction (AMI), few studies have described national, real-world trends in non-ST elevation myocardial infarction (NSTEMI) care in the emergency department (ED). We aimed to describe the characteristics, management, and outcomes of NSTEMI.

Methods

A prospective single-center study enrolled 40 NSTEMI patients in Alshaab Teaching Hospital during the period from May to July 2021. Data regarding demographics, medical history, clinical presentations, laboratory investigation, Killip classifications, electrocardiography (ECG), echocardiogram, diagnostic coronary angiography (CAG), management strategies, medications used, and 30-days outcomes were collected.

Results

Among 40 patients, NSTEMI was common in the age groups from 56 to 70 years (60%) and males (67.5%; p=0.002). Diabetes (n=24; 60%) and hypertension (n=20; 50%) were the major cardiovascular disease (CVD) risk factors. In most of the cases, 29 (72%) had a late presentation (>6 hours; p=0.0001). In Killip classifications, 36 (90%) patients were Killip class I and four (10%) were Killip class II (p=0.005). No patients underwent risk score assessment during a hospital stay. All patients had sinus rhythm in ECG and 28 (70%) had T-wave inversion. An echocardiogram was performed for 36 (90%) patients, among them six (16.7%) patients had LV systolic dysfunction (p=.003). The median ejection fraction was 52% (ranged from 25-75%). Diagnostic CAG was performed for 38 (95%) patients and a stent was inserted for 23 (58%) of them. The major final management strategy among our study group was PCI in 23 (58%) patients. All patients received aspirin, clopidogrel, parenteral anticoagulant, and ACEi/ARBs, 38 (95%) had statin, 28 (70%) were given PPI, and seven (17.5%) received diuretics. As for 30-day outcomes, all patients survived, but ten (25%) patients were readmitted, and no in-hospital or 30-days mortality occurred.

Conclusion

NSTEMI predominantly affected male and older patients. Most of them had a delayed presentation to ED. Hypertension and DM were the major risk factors. All patients were in sinus rhythm and the main ECG abnormality was a T-wave inversion. Most of the patients received standard NSTEMI protocol with exception of risk stratification. PCI was the major final management strategy used. Albeit no in-hospital or 30-days mortality occurred, 25% were readmitted.

## Introduction

Non-ST elevation myocardial infarction (NSTEMI) is a result of an acute imbalance between myocardial oxygen demand and supply, most commonly due to a reduction in myocardial perfusion. Classically, it is thought that NSTEMI patients ultimately have a diagnosis of a non-Q-wave MI; however, 25% of patients with NSTEMI and elevated biomarkers go on to develop Q-wave MI in the weeks to follow [1].

Non-ST elevation acute coronary syndrome includes a clinical spectrum that ranges from unstable angina to NSTEMI. Nevertheless, it is recognized that this broad spectrum of clinical presentations and outcomes results from common underlying pathophysiology, with atherosclerotic plaque disruption and differing degrees of associated thrombosis and distal embolization. While patients with NSTEMI, in comparison with those with ST-segment elevation MI (STEMI), have a greater prevalence of early culprit coronary artery patency, they are also at higher risk of recurrent ischemic events [2]. Patients presenting with chest pain or discomfort with suspected ACS require urgent evaluation. The clinical spectrum of NSTEMI may range from patients free of symptoms at presentation to individuals with ongoing ischemia, electrical or hemodynamic instability due to large myocardium in jeopardy, or cardiac arrest secondary to malignant ventricular ischemia [1]. 

Treatment for acute myocardial infarction (AMI), which includes STEMI and NSTEMI, is often started in the emergency department (ED). About more than 90% of people with AMI receive emergency care [3]. Although 60% of patients undergo same‐hospital admission, substantial portions are transferred between hospitals via the ED [4]. Clinical outcomes from NSTEMI may be enhanced through the adherence to guideline-indicated treatments comprising of evidence-based pharmacological therapies and invasive coronary processes [5,6]. Several cohort studies have revealed that improving compliance with evidence-based interventions decreased the hazard of death after NSTEMI. Though, between and within European country variation in the delivery and outcomes from NSTEMI recommended that the possibility to diminish the prevalence and incidence of cardiovascular disease (CVD) has not been understood [6-8]. Measuring documented standards of care is a procedure by which geographic differences in the use of guideline-indicated treatments for NSTEMI may be addressed and, therefore, cardiovascular outcomes improved [8,9].

Recently, the European Society of Cardiology (ESC) reported the results of two programs of work that are central to the management and quality improvement of patients with NSTEMI. The first document, the ‘2016 ESC guideline for the management of acute coronary syndromes in patients without persistent ST-segment elevation’, sets out the evidence-based road map for the optimal care of patients with NSTEMI. The second document, entitled ‘Quality indicators for acute myocardial infarction: A position paper of the Acute Cardiovascular Care Association’, details across seven domains and 20 indicators specific quality assessment indicators that include evidence-based process measures mapped to the 2017 NSTEMI guidelines and non-evidence-based dimensions important for quality improvement [10]. External validation studies in the United Kingdom (UK) and France propose that most of the Quality Indicators (Qis) were meaningfully contrariwise correlated with 30-day and -year mortality that is, the greater accomplishment of the ESC standards of NSTEMI care is related to favorable clinical outcomes [11,12]. 

Mortality rates for NSTEMI are high and survival worse than that for STEMI. The incidence of NSTEMI, which is already higher than for STEMI, is increasing with the aging and co-morbid European population [13-16]. Moreover, the prevalence and dissimilarity of NSTEMI care and outcomes continue notwithstanding considerable enhancements in its management [17-20].

Non-ST elevation myocardial infarction

NSTEMI is an acute ischemic event causing cardiomyocyte death by necrosis in a clinical setting consistent with acute myocardial ischemia [21]. The leading symptom that initiates the diagnostic and therapeutic cascade in patients with suspected ACS is chest pain but to make a diagnosis of NSTEMI, one major criterion is the typical rise and gradual fall in cardiac biomarkers (troponin or CKMB) in addition to one or more of the following [21]: (i) symptoms of ischemia; (ii) electrocardiography (ECG) changes; (iii) imaging evidence of new or presumed new loss of viable myocardium or regional wall motion abnormality; (iv) intracoronary thrombus detected on angiography or autopsy.

Research justifications

Cardiovascular diseases are the leading cause of death worldwide. Prompt recognition and initiation of appropriate management can save lives. NSTEMI is considered one of these top cardiac emergencies, carrying significant morbidity and mortality. This burden can highly be reduced by proper management and adherence to protocols and guidelines. The practice of evidence-based medicine is highly reliable on deep understanding and knowledge of demographics and patterns of the disease.

## Materials and methods

This is a prospective single tertiary cardiology center observational study conducted in the period from May to July 2021. All patients presented to Alshaab Teaching Hospital during the period from May to July 2021 with a confirmed diagnosis of NSTEMI according to the ESC definition of NSTEMI and based on the universal definition of acute myocardial infarction during the study period. Total coverage of all patients with NSTEMI fulfills the inclusion criteria of the study. This study included 40 patients during the study period.

Data collection was carried out by the principal investigator. Data were collected through structured questionnaires consisting of demographics, medical history, clinical presentations, laboratory investigation, Killip classifications, ECG, echocardiogram, diagnostic coronary angiography (CAG), management strategies, and medications used. Patients were followed up for 30-days to assess the outcomes in terms of surviving, readmission, and mortality. The study included all patients aged 18 years or above how their final diagnosis was NSTEMI presented to the center during the study period. We applied the Universal Definition of Acute Myocardial Infarction (type 1 MI) in the recruitment process. We only excluded the patients who refused to participate or do not fulfill the inclusion criteria.

Demographic data, clinical presentation, duration of symptoms, risk factors, laboratory investigations: creatinine, hematocrit, vital signs, Killip classifications, baseline ECG, echocardiogram, diagnostic CAG, final management strategies, medications used, inpatient mortality, and 30 days outcomes data were all included and reviewed for the targeted population during the study period.

Data analysis

Data were analyzed by using a computer program Statistical Package for Social Sciences (SPSS V. 21.0, IBM Corp., Armonk, NY). The analyzed data are presented in tables and figures designed by Microsoft Excel 2010.

Ethical approval

Ethical approval was obtained from Sudan Medical Specialization Board (SMSB) after being approved by the National Research Ethics-review Committee as part of the EURObservational Research Programme, NSTEMI Registry No. 2-4-19. Hospital administration’s approval and informed consent from patients were obtained as well. Data used anonymously by using identity numbers instead of names is ordered to protect the patient’s identity and kept secure in a separate file. No reference to any individual participant was made in study reports. Subject identities were known only by the study staff.

## Results

**Figure 1:**
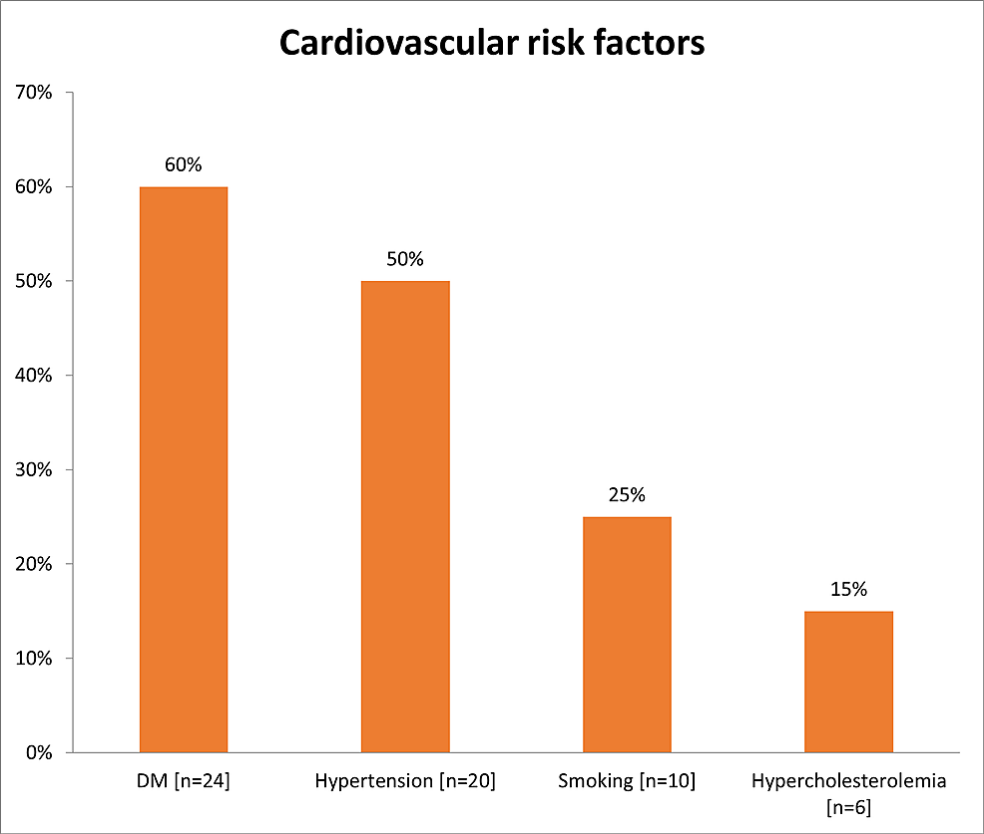
The distribution of risk factors for all patients DM: diabetes mellitus

**Figure 2:**
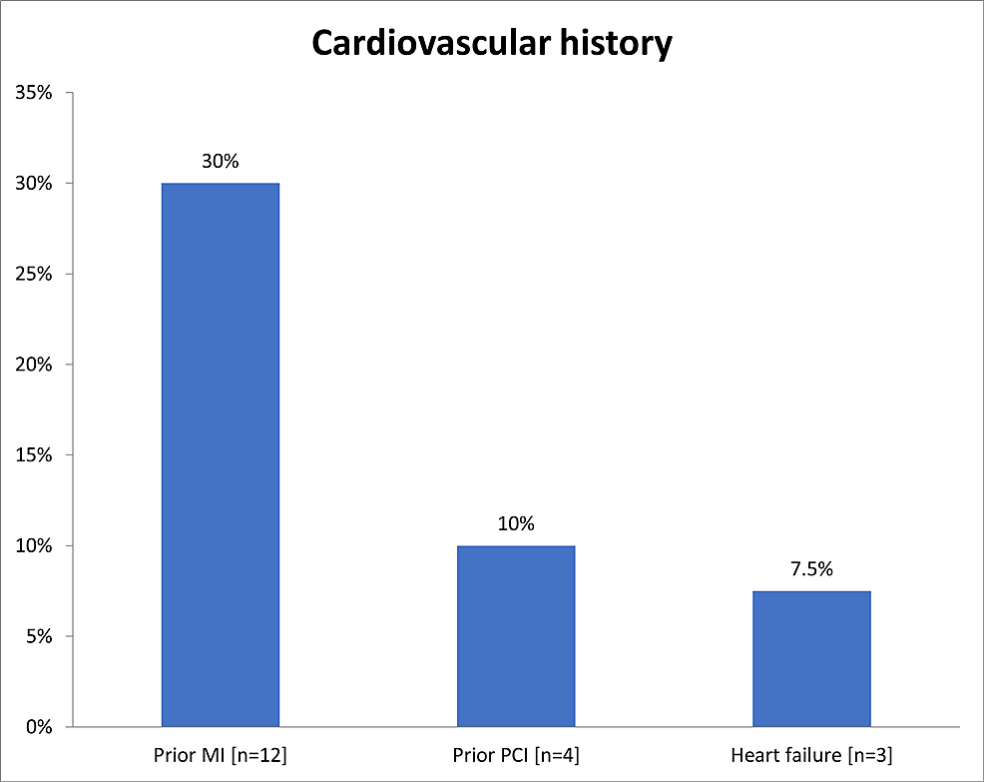
The cardiovascular history among the targeted population MI: myocardial infarction, PCI: percutaneous coronary intervention

**Figure 3:**
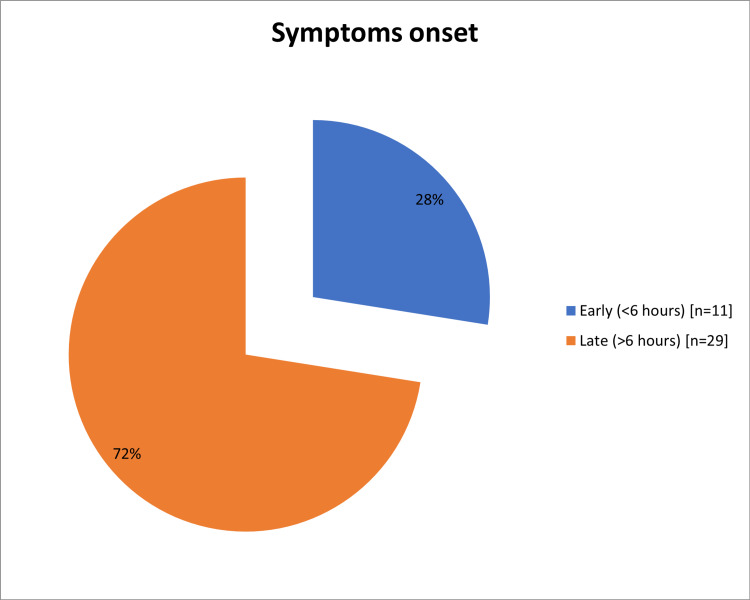
The onsets of symptoms for the study population (onset of chest pain)

**Figure 4:**
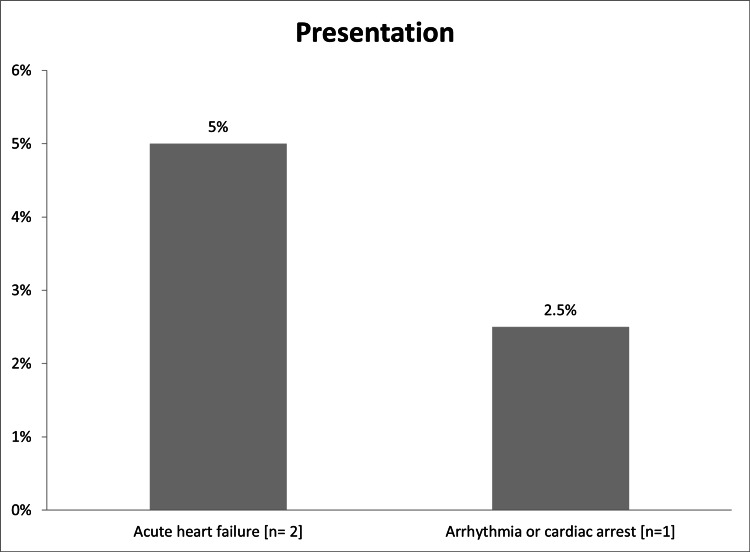
The clinical presentations of NSTEMI patients (N=40) NSTEMI: non-ST elevation myocardial infarction

In total, this study enrolled 40 NSTEMI patients, 27 (67.5%) were males and 13 (32.5%) were females, and most of the 24 (60%) were aged from 56 to 70 years (Table 1). Diabetes (n=24; 60%) and hypertension (n=20; 50%) were the major CVD risk factors encountered (Figure 1). Around 12 patients (30%) had prior MI, four (10%) had prior PCI, and three (7.5%) patients had a history of heart failure (Figure 2). Most of the patients had heart rate ranged from 60 to 100 bpm (n=37; 92.5%), systolic blood pressure ranged from 90 to 140 mmHg (n=33; 82.5%) and diastolic blood pressure ranged from 60 to 90 mmHg (n=34; 85%) (Table 2). Interestingly, 29 (72%) had late symptoms onset (>6 hours) and 11 (28%) had early-onset (<6 hours; Figure 3). Two patients (5%) presented with acute heart failure and one (2.5%) with arrhythmia or cardiac arrest (Figure 4).

**Table 1:** The demographic characteristics of the study population

	N	%
Gender; M:F	2:1	
Male	27	67.5
Female	13	32.5
Age (years)		
41–55	10	25.0
56–70	24	60.0
>70	6	15.0

**Table 2:** Heart rate and blood pressure for all patients HR: heart rate, BP: blood pressure, SBP: systolic blood pressure, DBP: diastolic blood pressure

	N	%
HR (bpm)		
<60	1	2.5
60–100	37	92.5
>100	2	5.0
SBP (mmHg)		
<90	1	2.5
90–140	33	82.5
>140	6	15.0
DBP (mmHg)		
<60	1	2.5
60–90	34	85.0
>90	5	12.5

**Figure 5:**
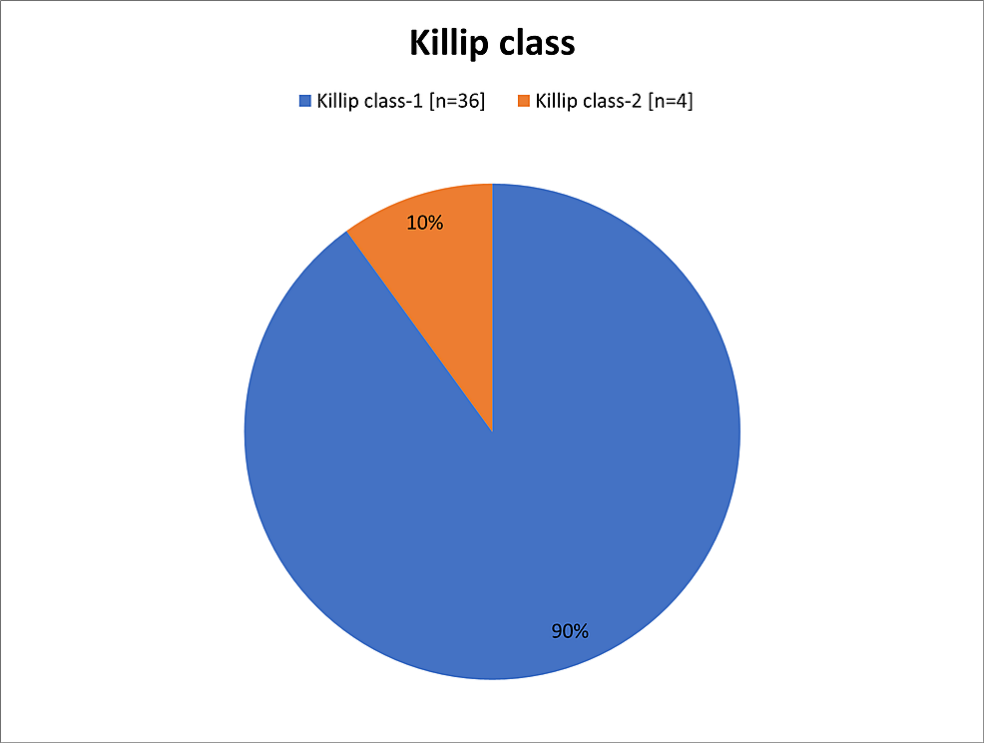
The Killip classifications of the study population

When we applied Killip classifications to the study population, we found around 36 (90%) patients were Killip class I, and the remaining four (10%) patients were Killip class II (Figure 5). With regard to the laboratory investigations, the vast majority of the subjects 38 (95%) had creatinine levels ranged from 0.8 to 1.9 mg/dl, and all of them (i.e., 40 patients) showed positive troponin. The mean of hematocrit was 38±5% and ranged from 32% to 45% (Table 3). As shown in Table 4, no patients in this study underwent risk score assessment during a hospital stay. All patients had sinus rhythm in their baseline ECG with 28 (70%) patients had T wave inversion, 10 (25%) had ST depression (5 in lateral lead, 4 in anterior and 1 in inferior lead), and one (2.5%) patient had right bundle branch block (RBBB; Table 5).

**Table 3:** The troponin and creatinine levels for the study population

	N	%
Creatinine (mg/dl)		
0.4–0.79	2	5.0
0.8–1.9	38	95.0
Troponin (positive)	40	100
Hematocrit; mean ± SD (min-max)	38±5 (32–45)	

**Table 4:** The risk score assessment performance among the study population

	N	%
Risk score performed during a hospital stay		
Yes	0	0.0
No	40	100

**Table 5:** Summary of the baseline electrocardiography findings ECG: electrocardiography, RBBB: right bundle branch block

	N	%
Baseline ECG		
Sinus rhythm	40	100
T-wave inversion	28	70
ST depression	10	25
In lateral lead	5	12.5
In anterior lead	4	10.0
In inferior lead	1	2.5
RBBB	1	2.5

**Figure 6:**
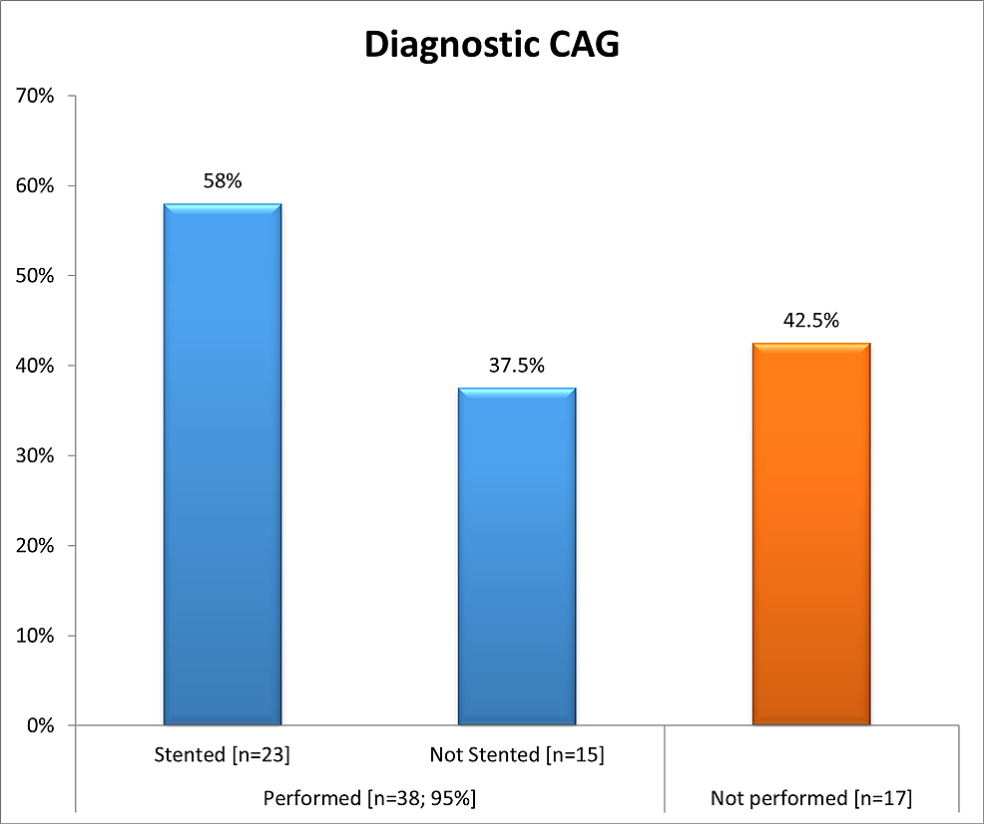
The diagnostic coronary angiography among NSTEMI patients (N=40) CAG: coronary angiography, NSTEMI: non-ST elevation myocardial infarction

**Figure 7:**
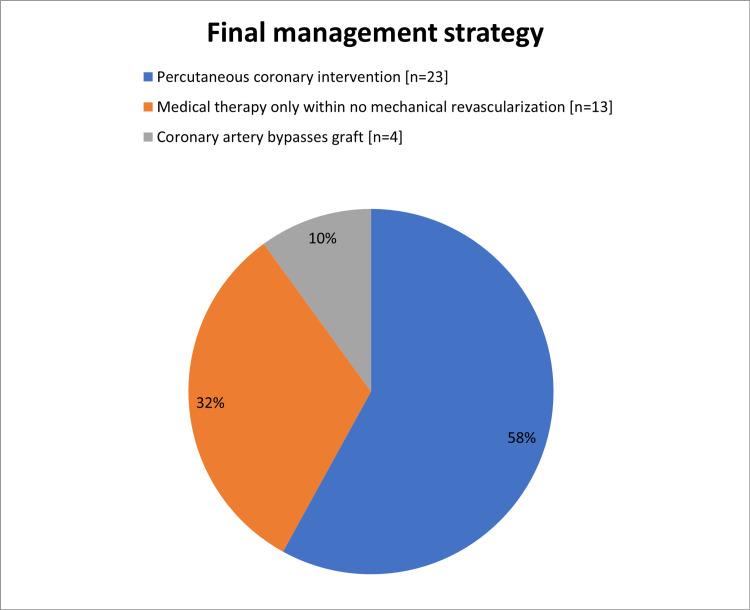
Management strategies

**Figure 8:**
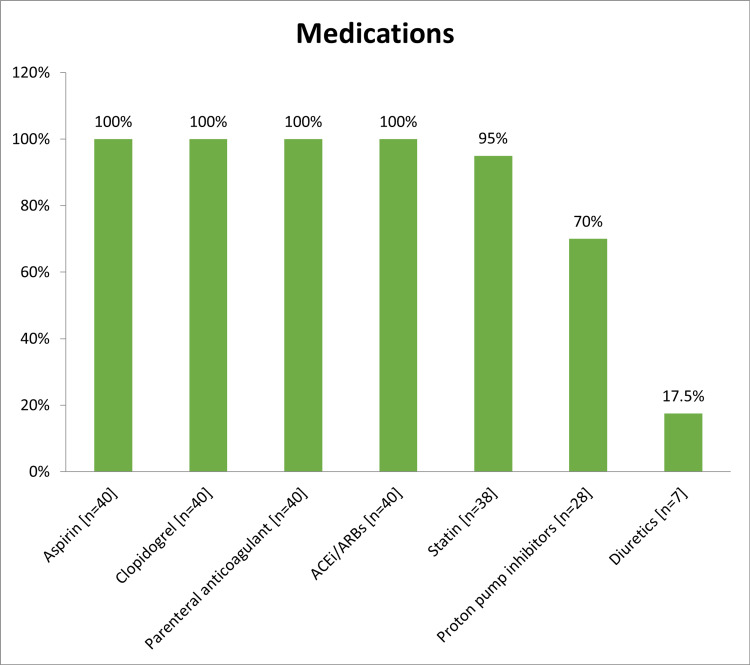
The medications received

**Figure 9:**
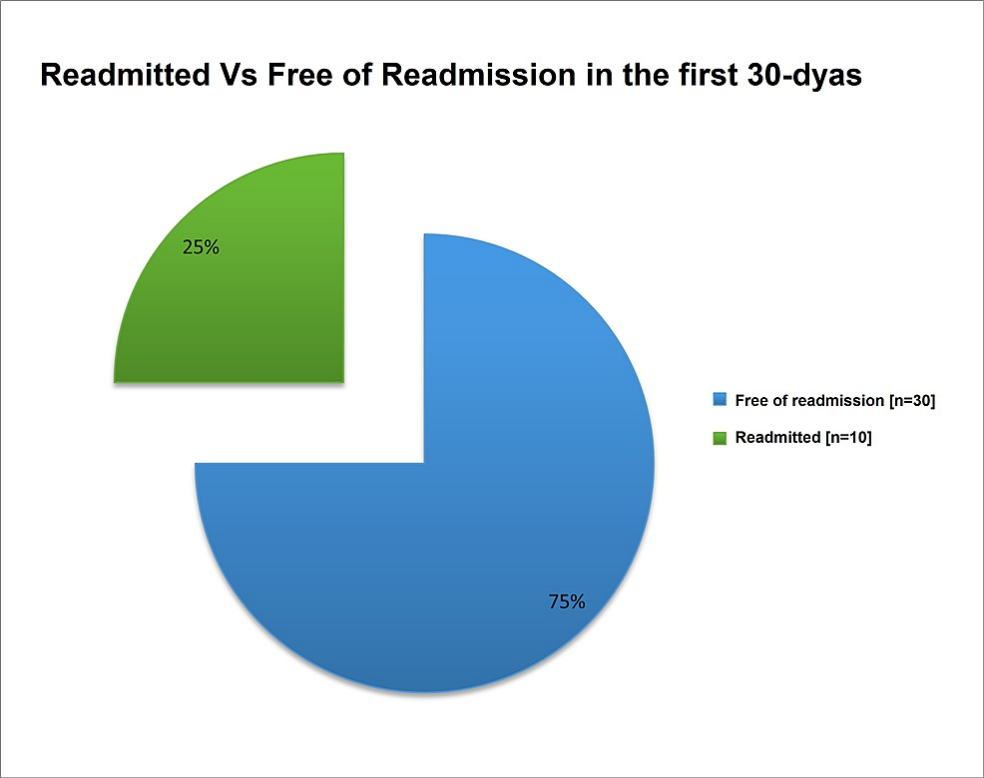
Readmitted versus free or readmission

All of the study population had been offered an echocardiogram, however, only 36 out of 40 patients had echocardiography done during the hospital stay. Among them, six (16.7%) patients had LV systolic dysfunction (mild in two patients, moderate in three patients, and severe in one patient) with the median ejection fraction was 52% (ranged from 25% to 75%), and most of them 31 (86.1%) had EF levels >50% (Table 6). Diagnostic CAG was performed for 38 (95%) patients, 23 (58%) of them stented and 15 (37.5%) were not (Figure 6). The major final management strategy among our study group was PCI in 23 (58%) patients followed by medical therapy only without mechanical revascularization in 13 (32%) and coronary artery bypass graft in 4 (10%) patients (Figure 7). All patients received (n=40; 100%) aspirin, Clopidogrel, Parenteral anticoagulant outside the cath lab, and ACEi/ARBs. Moreover, 38 (95%) patients received statin, 28 (70%) PPI, and 7 (17.5%) received diuretics (Figure 8). There is overall good adherence to the 2017 ESC Guideline with exception of risk stratification (0%) during a hospital stay. All patients (n=40; 100%) were alive during the hospital stay and no in‐hospital mortality was found (Table 7). Regarding 30-days outcomes, 30 (75%) patients were discharged from the hospital, and 10 (25%) patients were readmitted (Figure 9).

**Table 6:** The echocardiography findings of all patients

	N	%
Echocardiogram performed during hospital stay (Yes)	36	90.0
Category of LV systolic dysfunction (N=36)		
Normal	30	83.3
Mild	2	5.6
Moderate	3	8.3
Severe	1	2.8
EF (%); (n= 36); median (min-max)	52 (25–75)	
<30	1	2.8
30–39	1	2.8
40–49	3	8.3
50–59	14	38.9
60+	17	47.2

**Table 7:** The in-hospital mortality

	N	%
In‐hospital mortality		
Yes	0	0.0
No	40	100

## Discussion

The aim of this study is to describe the characteristics, management, and outcomes of NSTEMI in 40 patients attending Alshaab Teaching Hospital. In this study, NSTEMI predominantly affected patients aged from 56 to 70 years (60%), with a male-to-female ratio (2:1). These findings were in agreement with Sudanese studies of Mirghani et al. [22] and of Ahmed et al. [23], where MI was reported mainly among males and patients aged above 50 years. Also, Chung and Ying reported NSTEMI was frequent among males (M:F=1.9:1) and older patients (mean age=64.3±13.3 years) [24]. In the same line, Saman and Arnoud noticed that NSTEMI was common in males (78%) and old patients (mean age=67 ± 12 years) [25].

Diabetes (n=24; 60%) and hypertension (n=20; 50%) were the major CVD risk factors encountered. These observations were in accordance with a Spanish study in which hypertension, diabetes, and dyslipidemia were the major risk factors found among NSTEMI patients [26]. However, Hall et al. reported smoking (ex or current) as the chief risk factor of NSTEMI [6]. In general, as reported in the review of Ahmad and Mohammed, risk factors that increase the likelihood of NSTEMI includes, hypertension, DM, family history of CAD, smoking, hyperlipidemia, renal insufficiency, and the previous manifestation of CAD as well as peripheral or carotid artery disease [1]. On observations, most of the patients had normal heart rate (92.5%), systolic blood pressure (82.5%), and diastolic blood pressure (85%). Similar results were noticed by Hall et al. [6]. Most of the cases in this study (72%) had a late presentation (>6 hours). This could be explained by a lack of community awareness of the illness and insufficiency of the prehospital services. Similarly, among the Sudanese population, Mirghani et al. reported 64% of patients had a late presentation [22]. However, lowered duration between chest pain onset and ED arrival (median= 4 hours) was reported by Amsterdam et al. in Tunisia [27,28]. The present study showed that 36 (90%) patients were Killip class I and the remaining 4 (10%) patients were Killip class II. These findings were comparable to the study of Polonski et al. who reported 87% of NSTEMI patients had Killip classes I and II [15].

As recommended by ESC [10] and AHA guidelines [27], initial risk stratification is important in NSTE-ACS to predict outcomes and treatment selection by directly visualizing treatable targets in higher-risk individuals, since the benefit of intensive therapies varies with risk. In this study, no patient underwent risk score assessment during a hospital stay; this calls for the emerging use of scoring tools such as GRACE and TIMI in our protocol. ECG changes are one of the criteria in the diagnosis of NTEMI; in the current study, all patients (i.e. 40) had sinus rhythm. Consistently, in the study of Polonski et al., 91.2% of patients had sinus rhythm [15]. The main ECG abnormality found among our study groups was T-wave inversion (70%). As reported in the 2016 ESC guideline, T-wave flattening or inversion are major ECG changes in NSTEMI [10]. Also, in the study of Chung and Ying, T-wave abnormalities (65.6%) were the commonest ECG changes among NSTEMI [24]. According to the recommendations of the 2017 ESC guideline, an echocardiogram should be performed on all patients during their hospital stay. In our study, 90% of patients underwent echocardiograms. Among those, 16.7% had LV systolic dysfunction (mild in two patients, moderate in three patients, and severe in one patient). The median ejection fraction was 52% (ranged from 25% to 75%) and 31 of the patients (86.1%) had EF levels >50%. These findings were in agreement with National Heart, Lung, and Blood Institute Dynamic Registry by Abbott et al. in which the median EF was 51% [29].

This study showed that diagnostic CAG was performed for 38 (95%) patients and a stent was inserted for 23 (58%) of them. Our findings were sharply similar to the study of Rogers et al. who reported 57.4% of patients had inserted stent [30], higher than the study of Polonski et al. (29%) [15], and lower than that reported by Saman and Arnoud (78%) [25]. These variations might be due to differences in protocols between studies. The major final management strategy among our study group was PCI in 23 (58%) patients. PCI is the preferred reperfusion therapy if it can be performed by an experienced cardiologist within 72 hours of the first medical contact [24]. Our findings were greater than the Sudanese studies of Mirghani et al. (20.4%) [22] and Polonski et al. (23.1%) [15]. In other revascularization methods, CABG was received by 10% of our subjects. A comparable rate was reported by Saman and Arnoud who found 17% of NSTEMI patients received CABG [25]. All patients received (n=40; 100%) aspirin, Clopidogrel, Parenteral anticoagulant outside the cath lab, and ACEi/ARBs. These findings were in agreement with the recommendation of ESC and AHA guidelines [10,27]. In respect to 30-days outcomes, all patients survived but 10 (25%) patients were readmitted due to symptoms of angina. No in-hospital or 30-days mortality occurred, which is indicating there has been improvement in NSTEMI mortality rates over time. However, previous studies reported that, among patients with NSTEMI, in‐hospital and 30‐day mortality rates of between 5.2% and 13.1% and between 7.6% and 17.0%, respectively, have been reported [31,32]. In registries of Yeh et al. [31] and McManus et al. [32], the in‐hospital mortality rate was 7.1% in 1994 and 5.2% in 2006 [31], and from a 30‐day mortality rate was 10.0% in 1999 and 7.6% in 2008 [32].

## Conclusions

We conclude that NSTEMI predominantly affected male and older patients, with a delayed presentation to ED. Hypertension and DM were the major risk factors. All patients were in sinus rhythm and the main ECG abnormality was a T-wave inversion. Most of the patients received standard NSTEMI protocol with exception of risk stratification. PCI was the major final management strategy used. No in-hospital or 30-days mortality occurred. We recommend that the initial risk stratification of all NSTEMI patients should be used and clearly documented. Sudan needs ACS national registries (for both types STEMI and NSTEMI). Education of the general population about ischemic heart disease and improving prehospital services would decrease the time to presentation. Finally, more prospective studies with a larger sample sizes and longer follow-up periods are needed.
